# Quantifying and Valuing Potential Climate Change Impacts on Coral Reefs in the United States: Comparison of Two Scenarios

**DOI:** 10.1371/journal.pone.0082579

**Published:** 2013-12-31

**Authors:** Diana R. Lane, Richard C. Ready, Robert W. Buddemeier, Jeremy A. Martinich, Kate Cardamone Shouse, Cameron W. Wobus

**Affiliations:** 1 Stratus Consulting Inc., Boulder, Colorado, United States of America; 2 Department of Agricultural Economics and Rural Sociology, The Pennsylvania State University, University Park, Pennsylvania, United States of America; 3 Kansas Geological Survey, Lawrence, Kansas, United States of America; 4 U.S. Environmental Protection Agency, Climate Change Division (6207-J), Washington, D. C., United States of America; Bangor University, United Kingdom

## Abstract

The biological and economic values of coral reefs are highly vulnerable to increasing atmospheric and ocean carbon dioxide concentrations. We applied the COMBO simulation model (COral Mortality and Bleaching Output) to three major U.S. locations for shallow water reefs: South Florida, Puerto Rico, and Hawaii. We compared estimates of future coral cover from 2000 to 2100 for a “business as usual” (BAU) greenhouse gas (GHG) emissions scenario with a GHG mitigation policy scenario involving full international participation in reducing GHG emissions. We also calculated the economic value of changes in coral cover using a benefit transfer approach based on published studies of consumers' recreational values for snorkeling and diving on coral reefs as well as existence values for coral reefs. Our results suggest that a reduced emissions scenario would provide a large benefit to shallow water reefs in Hawaii by delaying or avoiding potential future bleaching events. For Hawaii, reducing emissions is projected to result in an estimated “avoided loss” from 2000 to 2100 of approximately $10.6 billion in recreational use values compared to a BAU scenario. However, reducing emissions is projected to provide only a minor economic benefit in Puerto Rico and South Florida, where sea-surface temperatures are already close to bleaching thresholds and coral cover is projected to drop well below 5% cover under both scenarios by 2050, and below 1% cover under both scenarios by 2100.

## Introduction

Coral reefs are highly diverse and productive ecosystems, providing valuable ecosystem services including recreation and tourism, fish habitat, and coastline protection. The net annual benefits of coral reefs globally have been estimated at approximately $33.6 billion per year, with tourism and recreation accounting for $10.8 billion (all values adjusted to US 2007$) [1]. An analysis of economic values of coral reefs in the Caribbean region estimated annual net benefits of $2.6 billion for tourism, $898 million to $2.7 billion for shoreline protection, and $376 million for coral reef fisheries (values are US 2007$; [2] as cited in [3]).

Overfishing, pollution, and habitat destruction are factors that are well-known to contribute to declines in coral reef cover and quality [4]. In recent years, it has become clear that greenhouse gas (GHG) emissions and climate change pose grave threats to coral reefs from the combined effects of elevated sea-surface temperatures (SSTs) that increase the risk of coral bleaching and rising atmospheric carbon dioxide (CO_2_) levels that result in increased ocean acidification, which affects the process of coral growth [5], [6], [7], [8], [9], [10].

Over the past 30 years, coral reef bleaching events have increased in both frequency and magnitude [10], [11], [12], [13]. The most extreme coral bleaching and mortality recorded in Caribbean and Atlantic coral reefs occurred in 2005, the warmest year of the global surface temperature record at that time [14]. In addition to global SSTs increasing by 0.67°C over the past century, ocean pH has already fallen by 0.1 unit and is projected to continue to decrease if future CO_2_ emissions continue on their current trajectory [15]. As pH declines, it is more energy-intensive for marine calcifying organisms, such as reef-building corals, to form biologically created (‘biogenic’) calcium carbonate that is the ‘backbone’ of coral structures [16]. The increase in ocean acidity projected to occur by 2050 is 100 times faster than any change that has been experienced in the oceans over the last 20 million years [17].

As the threats of climate change to human and natural systems have become more apparent, policymakers have focused on developing a better understanding of the potential benefits of reducing GHG emissions. Here, we focus on estimating the benefits related to coral reefs from a GHG mitigation policy. In particular, we project future reef cover and the economic values generated by coral reefs for a GHG emissions mitigation scenario that represents international implementation of policies to reduce global emissions, and compare this reduced emissions scenario to established projections for a ‘business-as-usual’ (BAU) emissions scenario [18]. The analysis is limited to United States coral reefs in Hawaii, South Florida, and Puerto Rico. To conduct our analysis, we used the COMBO (COral Mortality and Bleaching Output) model, which integrates the impacts of future climate change (i.e., thermal and ocean chemistry effects) on coral growth and mortality to estimate changes in coral cover over time in different locations [19], [20]. We then developed economic values for recreational services from and existence values for coral reefs based on published economic estimates from studies conducted at a range of coral reef sites, an approach known as ‘benefit transfer,’ and project how those values would differ between the BAU and the GHG emissions mitigation scenarios.

## Methods

### 2.1 Overview of Model

The COMBO model has been described in multiple previous studies [19], [20].The first presentation of the COMBO model [19] was primarily a model description paper, with preliminary results presented for Hawaii. The second presentation of the COMBO model [20] focused only on climate projections for the eastern Caribbean. These two manuscripts did not contrast the impacts of climate change across three separate regions, as we are doing here. In addition, neither of the two previous manuscripts included economic projections, which are described in further detail below. A modified version of COMBO is now available as open-source software code developed to run using the software program Matlab and available from https://sourceforge.net/projects/combocoralreef/, together with input files and default parameters. The description that follows briefly overviews the COMBO model as context for the modeling results presented here. The model provides estimates of changes in coral cover from 2000 to 2100 at monthly time steps, with results summarized and presented annually. Changes in coral cover are indicative of the health of coral communities and are calculated as the net difference between the rates of growth (including both growth of individuals and recruitment of new corals) and the rates of mortality from all causes.

The model consists of two interacting modules: a long-term change module and an episodic event module. The long-term change module calculates changes in coral cover over time in response to long-term changes in ocean chemistry and average SST. Superimposed on these long-term changes, the episodic event module calculates the impacts on coral cover of episodic mortality events caused by severe coral bleaching. During years when no bleaching mortality event occurs, coral cover increases or decreases based on estimated growth and mortality rates calculated in the long-term change module. When a bleaching-induced mortality event occurs, coral cover is reduced by the level of mortality associated with that event. The model then continues the simulation, with annual growth and mortality calculated by the long-term change module, until another bleaching-induced mortality event occurs.

The COMBO model is designed with a high degree of user flexibility to allow modeling of different coral reef conditions and scenarios. Parameters that can be varied by the user include the cumulative probability level that triggers a potential bleaching event (‘trigger probability’), the fraction of potential bleaching events that actually causes mortality (‘bleaching factor’), and the level of mortality associated with a bleaching event (‘mortality factor’). In addition, the user selects the future temperature model to use, and the local historical record of temperatures. The default values used for the modeling parameters are provided in Table 1 and are consistent with values used previously in COMBO modeling of changes in coral reef cover [19], [20].

**Table 1:** Parameters used for modeling simulations.

	Value					
Parameter	Bleaching event #1	Bleaching event #2	Bleaching event #3	Bleaching event #4	Bleaching event #5	Bleaching event #6
Climate scenario	SCENGEN generated outputs for a reduced emissions (‘policy’) scenario and a BAU scenario, assuming a 3°C sensitivity for doubled CO_2_.					
Baseline growth and mortality	3% per year.					
Saturation state sensitivity	Moderate sensitivity: 20% decrease in growth per unit decrease in Ωa, relative to an assumed maximum growth rate at Ωa = 4.6.					
Trigger probabilities	0.5	0.5	0.5	0.5	0.5	0.5
Bleaching factors	0.5	0.4	0.3	0.3	0.3	0.3
Mortality factors	0.3	0.4	0.5	0.5	0.5	0.5
Threshold temperatures for bleaching events – Hawaii	28.50°C	28.75°C	29.00°C	29.25°C	29.50°C	29.75°C
Threshold temperatures for bleaching events – Puerto Rico	29.5°C	29.7°C	29.9°C	30.1°C	30.3°C	30.5°C
Threshold temperatures for bleaching events – South Florida	30.2°C	30.4°C	30.6°C	30.8°C	40.0°C	40.2°C
Historical SST data source (used for intra-annual and interannual temperature distributions)	For 1950–1980: SST data available in a monthly 2° area grid [54], [55]. Also known as ERSST.v2 (referred to as the R2 dataset). For 1981–2008: SST data available in a monthly 1° area grid, from 1981 to 2008 [56], [57]. Also known as the ‘Reynolds and Smith Optimum Interpolation version 2’ dataset, or OI.v2 (referred to as the R1 dataset).					

In the episodic event module, bleaching is predicted on the basis of heat-dose estimates (degree-heating-weeks or degree-heating-months), which are calibrated against local temperature and bleaching records. Heat doses calculate the accumulated thermal stress on corals when SSTs exceed maximum expected summertime temperatures. Modeled heat doses are derived from a record of historical temperature variance superimposed on the rising temperature. Bleaching thresholds are identified as the temperatures for the monthly time step that would produce the critical heat dose. The threshold temperatures for the initial bleaching events were set to represent a heat dose as follows for each region. For Puerto Rico, the initial threshold temperature was set at 29.5°C, which represents a heat dose in the central range estimated for the 2005 bleaching event in the Eastern Caribbean, in general, and the Virgin Islands, in particular. The first modeled bleaching event would thus be approximately the same as the 2005 event in terms of dose. For Florida and Hawaii, the initial threshold temperatures were set at 30.2°C and 28.5°C, respectively, which are consistent with the heat doses associated with previous bleaching reports for those locations.

For subsequent bleaching events, the threshold temperatures were set progressively 0.2 degrees higher than the preceding threshold (∼2.6 additional degree heating weeks), based on the assumption that this is the approximate dose increment required to inflict comparable damage on a community from which the most vulnerable corals have already been removed [20]. Thus, this increase in threshold dose over time in the model reflects the fact that the most heat-sensitive corals are successively lost from the community after each bleaching mortality event, resulting in a simplified community that can sustain higher SSTs before bleaching.

In the original version of COMBO [19], [20], the model incorporated only three bleaching events. For South Florida and the Eastern Caribbean, three bleaching events are modeled to occur before 2020 for both the reduced emissions and BAU scenarios. Therefore, when conducting model runs from 2000 to 2100, the original model would not have included any bleaching events after 2020, which is not a realistic scenario. Accordingly, we revised the model so that three additional bleaching events could occur during the 21st century.

### 2.2 Valuation of Coral Reef Services

Coral reefs generate a wide variety of ecosystem services to humans including, but not limited to, providing habitat for aquatic species that are of value to humans, coastal structures that attenuate waves and protect shorelines from erosion, source materials for sand beaches, and recreational opportunities such as snorkeling and scuba diving. Although it is not possible to reliably estimate the values of all of these ecosystem services, data and tools are available to provide assessments of the value of some of these services. Here, we focus on two important categories of value: use values from reef-based recreation and existence values. The resulting calculated economic losses associated with declines in coral reef cover provide lower-bound estimates of the true losses that would be associated with all coral reef services.

#### 2.2.1 Reef-based recreation

The most recognizable use values generated by coral reefs are the benefits enjoyed by people who visit the reefs for recreation. The value of a reef visit to a person visiting the reef for recreation (the ‘recreationist’) is defined as the maximum additional amount of money the recreationist would be willing to pay to access the reef on that occasion, over and above the actual cost. As an illustration, consider a potential snorkeler who would pay up to $75 for a chance to snorkel on a coral reef. If the cost of visiting the reef is greater than $75, he or she will choose to do something else on that day. If the potential snorkeler faces actual access costs totaling $55, then he or she will engage in the activity, and enjoy a net benefit of $20 from the visit. This benefit is called the consumer surplus to the recreationist from the visit. It represents the net benefit to the recreationist of engaging in a visit to the reef instead of engaging in some other activity.

To generate a measure of consumer surplus per visit for reef-based recreation, we take a ‘benefit transfer’ approach, where benefit estimates measured in one area (the study site) are used to value benefits enjoyed in a different area (the policy site) [21]. The benefit transfer approach used here is called unit value transfer. In unit value transfer, an estimate of the value of one unit of resource use (in the current case, one visit to the reef by one recreationist on one day) is obtained from a study conducted at the study site, or an average estimate is calculated from several studies conducted at multiple study sites. The value of the change in the resource at the policy site is calculated by multiplying the transferred unit value (consumer surplus per reef visit) by the number of units of the resource affected (the change in the number of reef visits). This approach assumes that the value of a day of reef recreation at the study site(s) is the same as the value at the policy site. If reef quality, or the availability of substitute sites, or the characteristics of the reef users differ between the study site and the policy site, then the value per visit will likely differ as well, introducing error into the transfer. This approach also assumes that the value per recreation visit will remain constant as the reef resource declines. If declining reef quantity results in increased crowding at reef sites, the value per day could change [22].

A more advanced benefit transfer approach is value function transfer. In some cases, a source study will estimate a value function that describes how the value per unit of the resource varies depending on the nature of the resource or of the resource users. In such cases, the analyst can use the value function estimated at the study site to predict the unit value at the policy site. If no single study is available that has estimated a value function, it may be possible to estimate a value function based on unit value estimates from multiple source studies, an approach called meta-analysis. A value function, whether it comes from a single source study or is estimated using meta-analysis, will allow the analyst to account for differences in value between the study site(s) and the policy site that are related to measurable differences in the resource or its users. A challenge for conducting value function transfer is that the characteristics of the resource must be quantified and measured consistently across different study sites. For example, in the current application, because the consistent measures of the recreational quality of reefs are not available across different regions, it is not possible to estimate how consumer surplus per visit varies with reef quality. Because value functions that account for differences in the characteristics of reef resources are not available for U.S. reefs, we are restricted to using unit value transfer in this study. This restriction introduces potential errors, whereby reef values may be overvalued for some regions and undervalued for other regions.

The reliability of a benefit transfer, that is the percent error introduced by using transferred estimates instead of original estimates generated at the policy site, depends on the degree of similarity between the study site(s) and the policy site in terms of the resource, how the resource is used, the resource users, and the change in the resource being valued. When the study site and policy site have similar resources and users, transferred benefit estimates often fall within 20–40% of estimates generated at the policy site [23]. Benefit transfers tend to be more reliable when the study site and the policy site are located close to each other, and transfers conducted within a country tend to be more reliable than transfers conducted between countries [24], [25]. For this reason, we limit our analysis to only use source studies conducted in the United States and its territories that measured consumer surplus for reef-related recreation (Table 2).

**Table 2:** Review of estimated snorkeling and diving recreational values.

Study	Study region	Valuation method	Consumer surplus per day (US 2007$)
Leeworthy and Bowker [58]	Florida	Travel cost	$121.04
Park et al. [59]	Florida	Contingent valuation	$191.93
		Travel cost	$65.37
Cesar et al. [26]	Hawaii	Contingent valuation	$11.18^a^
Bhat [38]	Florida	Travel cost	$161.04
Johns et al. [27]	Florida	Contingent valuation	$14.91
Oh et al. [60]	Gulf of Mexico	Contingent valuation	$220.46
Estudios Técnicos [28]	Puerto Rico	Travel cost	$112.86
		**Average**	**$112.35**
		(95% confidence interval)	($58.41, $166.29)

^a^ . Average of values for residents, visiting snorkelers, and visiting divers.

We use the average value from all studies in Table 2, $112.35, as our measure of consumer surplus for a recreational visit to a coral reef in Hawaii or South Florida. We multiply this per-visit estimate of consumer surplus by estimates of the total number of reef visits in Hawaii [26], and Florida [27] to obtain total recreational use values for those two regions (Table 3). We chose to use the same average estimate of consumer surplus for both Hawaii and South Florida because the only consumer surplus estimate we located for Hawaii [26] reported a consumer surplus for reef-based recreation of around $11 per visit, which was less than 10% of the average estimate across the other studies. We believe the payment card methodology used in the Cesar et al. study in Hawaii [26] may have biased their estimate downwards. Thus, we use an average estimate across all U.S. studies for both Hawaii and South Florida instead of using a site-specific value that we believe has low reliability.

**Table 3:** Summary of measurable annual values of ecosystem services (US 2007$).

	Hawaii	Florida	Puerto Rico
**Recreational use value**			
Recreational visits (million)	15.5	18.2	n.a.
Recreational use value per visit	$112.35	$112.35	n.a.
**Total recreational use value (**$ **million)**	$**1,741**	**$2,039**	**n.a.**
(95% confidence interval)	($905, $2,577)	($1,060, $3,018)	
**Existence value**			
Population – 18 years or older (million)	0.92	3.89	2.91
Existence value per adult	$104.93	$104.93	$104.93
**Total existence value ($ million)**	**$96**	**$408**	**$305**
(95% confidence interval)	($90.7, $101.5)	($385, $431)	($288, $322)

Sources:

Recreational visits – Hawaii: Table 8.1 in Cesar et al. [26]. Florida: Table ES-5, natural reefs only, in Johns et al. [27]. Puerto Rico: estimates of total recreational use unavailable.

Recreational use value per visit – Average of seven studies conducted in Florida, Hawaii, Puerto Rico, and the Gulf of Mexico (see Table 2).

Number of adults – Hawaii: 2010 census. Florida: 2010 census (includes only Broward, Miami-Dade, Monroe, and Palm Beach counties). Puerto Rico: Adult population from p. 83 in Estudios Técnicos [28].

Existence value per person – Table 2, existence value, in Estudios Técnicos [28].

An estimate of total reef recreation in Puerto Rico is available for Puerto Rico residents [28], but not for nonresidents. In 2010, there were 1.4 million tourist arrivals in Puerto Rico [29]; however, the fraction of these visitors who participate in reef-related recreation is not known. For this reason, we do not present an estimate of the recreational value of reef resources in Puerto Rico.

#### 2.2.2 Existence values

Existence values are benefits that accrue to individuals independent of any direct or indirect use of the resource [30]. Individuals not using reefs may still hold positive values for maintaining reef extent and quality. These values could be motivated by concern for those who do use the reef (altruism), by a desire to preserve the ecosystem for potential future users of the reef (bequest values), or by concern for the reef resource itself (existence values) [31], [32]. An individual's existence value for a coral reef is defined as the amount of money the individual would be willing to pay to maintain the reef's quality and extent and to prevent loss of the reef.

We reviewed three studies that report existence value estimates for U.S. reefs. A study conducted in Hawaii [33] used a stated choice survey to estimate existence values for improvements in reef quality in Hawaii. The specific reef-quality improvements valued were an increase in reef health (e.g., more fish, less coral mortality due to algae growth) and repair to reefs damaged by ship collisions. Existence values were estimated for all U.S. residents, not just local residents. Unfortunately, the changes in reef quality valued in the Hawaii study cannot be directly compared to the changes in reef cover projected in this study, so the estimates of existence value from the Hawaii study cannot be used for the purposes of this study.

A study in Florida [34] conducted a thought experiment regarding the potential size of existence values for Florida coral reefs. That study did not estimate existence values for reefs, but rather speculated about how large existence values for reefs might be, based on estimates of existence values for other, similar resources. Because the existence value estimates presented in that study are not specific to coral reefs, we do not use those values in the benefit transfer exercise.

A study conducted in Puerto Rico [28] used a contingent valuation survey to estimate existence values for coral reefs. The average amount that Puerto Rico residents would be willing to pay annually to avoid complete loss of the coral reef was $104.93 per adult (Table 3). Through scaling, described below, it is possible to use this estimate to value projected losses in reef cover. Thus, we chose to apply the Puerto Rico existence values to each of our modeling regions. Because average household income in Puerto Rico is lower than in Florida or Hawaii, this benefit transfer approach likely results in an underestimate of existence values for reefs in Hawaii and Florida.

Ideally, existence values for U.S. reefs would be measured for all U.S. residents. Unfortunately, estimates of existence values for changes in reef cover are not available for the general U.S. population. The Puerto Rico study [28] estimated existence values of residents of the same jurisdiction the reefs were located in. We therefore calculate the existence values associated with changes in reef cover only for local residents. For Puerto Rico and Hawaii, the existence value per adult was multiplied by the number of adult residents of Puerto Rico and Hawaii, respectively. For Florida, the per-adult value was multiplied by the number of adult residents of the four southern Florida counties that include coral reef resources (Broward, Miami-Dade, Monroe, and Palm Beach). This is a conservative approach, as U.S. residents living outside these three areas likely also hold existence values for reefs that are not included in the analysis.

The resulting recreational use value and existence value estimates are shown in Table 3. We do not present estimates of total value of reef resources for each region for two reasons. First, we do not value all ecosystem services provided by coral reefs. Second, existence values for a reef may not be independent of recreational use value [35]. As explained above, the following caveats must be stressed about the values presented in Table 3. First, recreation values for Puerto Rico are not presented because estimates of nonresident recreational use are not available. Second, estimated existence values for Hawaii and Florida are likely underestimated because they are based on an estimate from Puerto Rico, where average incomes are lower. Third, all estimates of existence values for all three regions are likely underestimated because they only include existence values of local residents.

In addition to the caveats made above, we point out that the values presented in Table 3 assume that coral reefs are the only resource that is changing over time. Other recreational resources that serve as substitutes for reef-based recreation, for example fresh- and salt-water fishing, will likely also change over time, in part as a response to climate change, affecting the value of reef-based recreation. Climate change, and climate change policies, will also influence the broader economy (and incomes) in ways that could affect both recreational and existence values for reefs [36].

#### 2.2.3 Estimating a decline in value as coral cover decreases

The recreational value of coral reefs is likely to be affected by the widespread loss of shallow coral reefs. Snorkeling activities are likely to decrease because snorkelers cannot switch to deeper reefs. Recreational diving is also likely to be affected because diving to deeper reefs requires additional training and equipment.

Two studies provide information on the relationship between recreational use value and reef health. In a contingent behavior study of divers and snorkelers on boats who had visited the Great Barrier Reef, tourists were asked how their future trips would change if the reef changed to a degraded state with an 80% decline in coral cover, a 70% decline in fish diversity, and a 30% decline in coral diversity [37]. The study found that there could be an 80% decline in future trips, from a mean of 2.82 trips to 0.56 trips, suggesting that reef visitation is directly proportional to coral cover. A study of reef visitation in the Florida Keys [38] found that a 100% increase in reef health would result in a 105% increase in consumer surplus, also suggesting an approximately proportional relationship between coral health and recreational use values.

Consistent with these findings, we assume that a given percent decrease in coral cover would cause an equal percentage decline in recreational value. A caveat should be noted. The recreational use value estimates presented in Table 2 are each developed for one reef area, under the assumption that reefs in other areas will remain unchanged. Our calculated recreational use values losses will underestimate the true losses from coral cover declines that occur in all three study regions, and presumably in other regions as well, because affected recreationists will have fewer substitution possibilities.

We are unaware of any studies that have looked at the loss of existence values of coral reefs in response to changes in coral reef cover. Therefore, we assumed that a percentage loss in reef cover would cause an equal percentage loss in existence value, consistent with our treatment of recreational values. However, we acknowledge that the relationship between coral cover and existence values could be nonlinear.

### 2.3 Other Coral Values Not Included in Our Analysis

Our study did not include several other categories of coral reef values in the overall analysis, including the commercial harvest of reef-related species and coastal protection. This section describes these values and the reasons they were excluded.

Humans use coral reefs through the commercial harvest of fish and invertebrates from the reef, or of fish that rely on reefs during some stage of their lifecycle. An annual dockside value of reef-related commercial fish harvest in Hawaii was estimated at $4.7 million, including both harvests for food and for the aquarium trade [26]. In 2006, the total annual commercial fishery harvest for eastern Puerto Rico was valued at $566,000 [28]. This latter estimate includes both reef-related fish and non-reef-related fish.

A decline in coral reef cover would likely reduce the productivity of reef-dependent fish stocks, and could result in decreased fish harvests, which could impact consumers and harvesters of reef-dependent fish. Because reef fisheries comprise a small proportion of total fish harvest, a decline in harvest from reef-dependent fisheries would have a small impact on U.S. fish consumers. Commercial harvesters could see declines in catch and incomes, but those declines are likely to be small relative to the recreational use values and existence values included in the analysis. First, the total value of commercial harvest is small relative to the values included in Table 3. Second, most reef-based fisheries are open access, so that commercial harvests are already depressed and incomes are already low [39]. As a practical matter, it is difficult to project the specific impact that the loss of coral cover will have on reef fish productivity. For these reasons, impacts on commercial fish harvests from changes in reef quality and extent are not included in this analysis.

Because of their location and structure, coral reefs can provide important wave attenuation and shoreline protection services, both because they protect the beach from erosion and because they provide coral sand to form beaches. The economic value of the shoreline protection benefit from reefs has been estimated for some site-specific case studies. For example, the shoreline protective value of coral reefs is estimated between $18 and $33 million for Tobago and between $28 and $50 million for St. Lucia [40]. However, similar studies are unavailable for Hawaii, Florida, and Puerto Rico, and differences in exposure and topography make the transfer of shoreline protection benefit estimates from one site to another unreliable. For these reasons, we did not attempt to measure the economic value of this ecosystem service.

Finally, we do not include projections of change in regional economic activity (e.g., recreational expenditures) in our estimates of future losses in economic value. As pointed out earlier, the value generated by reef recreation is the amount that a recreationist is willing to pay over and above their expenditures. Further, changes in reef-related recreational expenditures would not necessarily result in changes in total economic activity. For example, a visitor to Hawaii who previously spent money on a snorkeling or diving trip may instead spend money on a land-based activity, such as visiting a volcano, which could result in no net loss to the regional tourism economy.

### 2.4 Climate Scenarios

This analysis compares a BAU scenario with a hypothetical reduced emissions scenario. The BAU scenario is a path that GHG emissions are projected to follow if emissions continue on their current trajectory. Under the BAU scenario, U.S. GHG emissions are benchmarked to the U.S. Energy Information Administration's Annual Energy Outlook 2010 forecast, which reflects the Energy Independence and Security Act of 2007 and the American Recovery and Reinvestment Act of 2009. International emissions projections under this scenario are based on the EMF-22 baseline [18]. By 2100, atmospheric concentrations of CO_2_ under this BAU scenario will rise to 785 ppm, nearly three times pre-industrial levels.

The reduced emissions scenario is a hypothetical GHG emissions mitigation scenario that represents international implementation of policies to reduce global emissions. Under the reduced emissions scenario, two models (GCAM [18] and MAGICC [41]) were used to analyze how U.S. GHG targets, combined with international action, could affect global carbon dioxide equivalent (CO_2_e) concentrations and temperatures. U.S. GHG emissions are assumed to be consistent with targets included in the American Power Act of 2010, a comprehensive energy and climate bill proposed in the 111th Congress. The scenarios are grounded in analysis conducted for the American Power Act of 2010; however, the focus of this paper is not related to that analysis. The assumptions about international action are based on targets set at the July 2009 Major Economies Forum along with other assumptions regarding future emissions from developed and developing countries. Specifically, for non-U.S. Group 1 countries (i.e., Kyoto Protocol countries excluding Russia), the reduced emissions scenario assumes that GHG emissions follow a downward linear path from simulated Kyoto emissions in 2012 to 83% below 2005 emissions in 2050. Emissions in all other countries are assumed to continue on the BAU trajectory through 2025, at which time emissions are capped at 2015 levels. After 2025, emissions follow a linear, downward trend until emissions fall to 26% below 2005 emissions by 2050. In all countries, emissions are assumed to remain flat from 2050 through 2100. Atmospheric CO_2_ concentrations under this scenario rise to 427 ppm by 2100, and this trajectory is approximately comparable to the more well-known Intergovernmental Panel on Climate Change (IPCC) SRES-B1 scenario, which yields an approximate CO_2_e concentration of 600 by 2100 [42]. See Table 4 for a comparison of the two scenarios from 2000 to 2100.

**Table 4:** CO_2_ concentrations (ppm) for alternative emissions scenarios used in the model, for the years 2000–2100.

Scenario	2000	2030	2050	2075	2100
BAU	369	443	519	639	785
Reduced emissions	369	421	426	423	427

Future atmospheric CO_2_ and temperature projections, based on the above emissions scenarios, were obtained from the Model for the Assessment of Greenhouse-gas Induced Climate Change (MAGICC v. 5.3) and Scenario Generator (SCENGEN) [41]. MAGICC has been one of the climate models used by the IPCC to produce projections of global-mean temperature and precipitation. The global-mean temperatures from MAGICC are used by SCENGEN, which also uses the results of Atmosphere/Ocean General Circulation Models (AOGCMs), to create spatially explicit patterns of temperature change for a common 2.5° latitude/longitude grid. Temperatures obtained from SCENGEN are lower-atmosphere temperatures because SSTs are unavailable from the MAGICC/SCENGEN model. However, tests of these predictions against modeled future SST values have shown differences <0.2°C for the locations of interest [19].

MAGICC/SCENGEN provides information on the variability among the AOGCMs. Rather than use the results of a single AOGCM, MAGICC/SCENGEN allows the evaluation of the average over a number of models to increase the robustness of the estimates [43]. Reported changes in monthly temperature are averages of the values from nine models. Models included are designated by the following acronyms: BCCRBCM2, CCSM-30, ECHO-G, GFDLCM20, GFDLCM21, MPIECH-5, MRI-232A, UKHADCM3, and UKHADGEM. These models are described on the website for the Program for Climate Model Diagnosis and Intercomparison [44]. These nine models are representative models that were part of the general circulation model set used in IPCC's Fourth Assessment Report [45]. The primary basis for the model selection was their ability to simulate current climate patterns globally and over the coterminous United States, as reflected in the pattern correlation scoring feature within SCENGEN [41].

For each scenario, CO_2_ concentrations were obtained at five-year intervals; a linear interpolation was used to estimate annual values. We also obtained projected temperatures for the years 2000, 2020, 2030, 2050, 2075, and 2100 (expressed as degrees Celsius above the year 2000 temperature) and used linear interpolation to estimate annual values. In addition, both scenarios were run with the same ‘climate sensitivity’ value of 3°C, which means that the models assume that a prolonged doubling of the pre-industrial CO_2_ concentration of 280 ppm will result in a 3°C increase in global mean near-surface air temperature. This value of 3°C was the ‘most likely sensitivity’ reported in the Fourth Assessment Report [15]. Note that both CO_2_ concentrations and projected temperatures are higher under the BAU scenario than under the reduced emissions scenario, even though the sensitivity of climate to CO_2_ is the same between the two scenarios.

Scenarios were run using an ‘area smoothed’ output, where the value for any grid cell is calculated as the average of the given grid cell and the eight surrounding cells. Smoothing is useful for estimating climate changes at a specific latitude/longitude location. Because results for the individual 2.5°×2.5° cell in which the site is located are subject to more noise than a larger area surrounding the site, a nine-cell (7.5°×7.5°) area average is generally considered a more stable estimate of site changes than an individual cell [46].

### 2.5 Initial Estimates of Coral Cover

Coral reef cover data were assembled for the three regions (Puerto Rico, South Florida, and Hawaii), with the goal of approximating conditions in the year 2000. Data were compiled that measured the percent of area covered by hard corals in the field, using underwater survey techniques. Data from 1998 to 2003 were cross-checked for consistency with other data and accuracy of locational information, and then classified as either deep (i.e., >5 m depth) or shallow reefs. This study uses the same coral cover datasets that were used in a previous COMBO study [19].

For each region, there were fewer grid cells with coral reef cover data, compared to the number of grid cells where we had estimates of hardground area (potential coral). We had estimates of coral cover for 7 grid cells in Hawaii, 2 grid cells in Florida, and 2 grid cells in Puerto Rico. The modeling projections of coral cover decline are based solely on those grid cells where we had initial estimates of coral cover. We calculated an average across cells for our overall estimate of coral cover decline in each region (this average is not weighted because it already takes into account both the starting cover in a cell and the projected decline over time).

### 2.6 Calculation of Changes in Coral Reef Values over Time

As part of our analysis, we needed to estimate how coral reef value would decline across each region (i.e., Hawaii, Florida, Puerto Rico). As described above, we had estimates of coral cover based on field data for only a few of the grid cells that were modeled. For the valuation calculations, we needed a method to estimate an average decline in coral cover for all of the grid cells within a region, since different cells within each region had different amounts of starting coral cover and different projected trajectories of decline within COMBO. Given the lack of spatially comprehensive and up-to-date coral cover datasets, we obtained estimates of ocean hardground areas in each region for individual 1°×1° cells to serve as approximations of areas suitable for coral reef establishment (while acknowledging that these areas do not represent actual amounts of coral cover) [47]. We estimated hardground areas for 14 cells in Hawaii, 9 cells in Florida, and 6 cells in Puerto Rico by using primary data for the hardground area in Florida [47] and calculating the ratio to total benthic area in the 0–100 m depth range. This ratio was then combined with the regional benthic area data [47] to calculate approximate hardground areas for Hawaii and Puerto Rico. We calculated a weighted average of coral reef decline for each region (using the relative amount of hardground in each grid cell for weighting), and then multiplied this weighted average by the original (baseline) recreational and existence values for each region.

Annual values were discounted to present values in 2007 using a 3% discount rate, and summed over the period of 2000 to 2100 for each region. This was done for both the BAU scenario and the reduced emissions scenario. A base year of 2007 was selected for consistency with other ongoing and relevant analyses not discussed in this manuscript. The choice of base year affects the dollar value of our results, but not the relative differences between scenarios. The U.S. Interagency Working Group on Social Cost of Carbon [48] recently reviewed and considered the selection of discount rates for climate change impacts analysis. CO_2_ emissions are long-lived and the damages they incur are realized over many years, therefore raising issues regarding the choice of discount rate for intergenerational problems. To address this, the Working Group selected three discount rates: 2.5%, 3%, and 5% per year. For the analysis in this paper, we chose the Working Group's central value, 3%, which is also consistent with estimates provided in the economics literature and the Office of Management and Budget's Circular A-4 guidance [49] for the consumption rate of interest.

## Projected Climate Change Impacts: Results from Ecological and Economic Modeling

### 3.1 Timing of Bleaching Events

For South Florida and Puerto Rico, temperatures by 2000 have already surpassed the initial bleaching threshold. For these locations, there is a difference of just one or two years between the reduced emissions scenario and the BAU scenario in the projected dates of bleaching for the first three modeled bleaching events (Table 5). The reduced emissions scenario does project a more substantial delay in the timing of the sixth bleaching event (which is the last event simulated by COMBO) compared to the BAU scenario. In Florida, the reduced emissions scenario delays the sixth bleaching event by approximately 20 years, while in Puerto Rico the reduced emissions scenario delays the sixth bleaching event by approximately 40 years.

**Table 5:** Timing of six modeled bleaching events in each location^a^.

	South Florida		Puerto Rico		Hawaii	
Bleaching event	BAU	Reduced emissions	BAU	Reduced emissions	BAU	Reduced emissions^b^
Event #1	2001	2001	2001	2001	2032	2048
Event #2	2004	2005	2006	2007	2042	2068
Event #3	2014	2015	2017	2019	2050	2076
Event #4	2023	2026	2027	2031	2059	
Event #5	2031	2038	2034	2047	2067	
Event #6	2037	2055	2042	2081	2076	

^a^ Bleaching dates are calculated as averages across all modeled grid cells in each location.

^b^ Under the reduced emissions scenario, only 2 of 14 modeled grid cells in Hawaii experience three bleaching events by 2100.

In Hawaii, the earliest bleaching event in any single grid cell is projected to occur in 2016, with an average date for the first bleaching event of 2032 (Table 5). There also is a notable difference between the reduced emissions and BAU scenarios for the timing of all bleaching events. Under the reduced emissions scenario, the first two bleaching events are delayed by approximately 15–25 years compared to the BAU scenario. In addition, the number of bleaching events projected for Hawaii differs between the two scenarios. For the reduced emissions scenario, only two grid cells experienced a third bleaching event by 2100, while under the BAU scenario, all grid cells experienced six bleaching events by 2100.

### 3.2 Changes in Coral Cover

**Figure 1:**
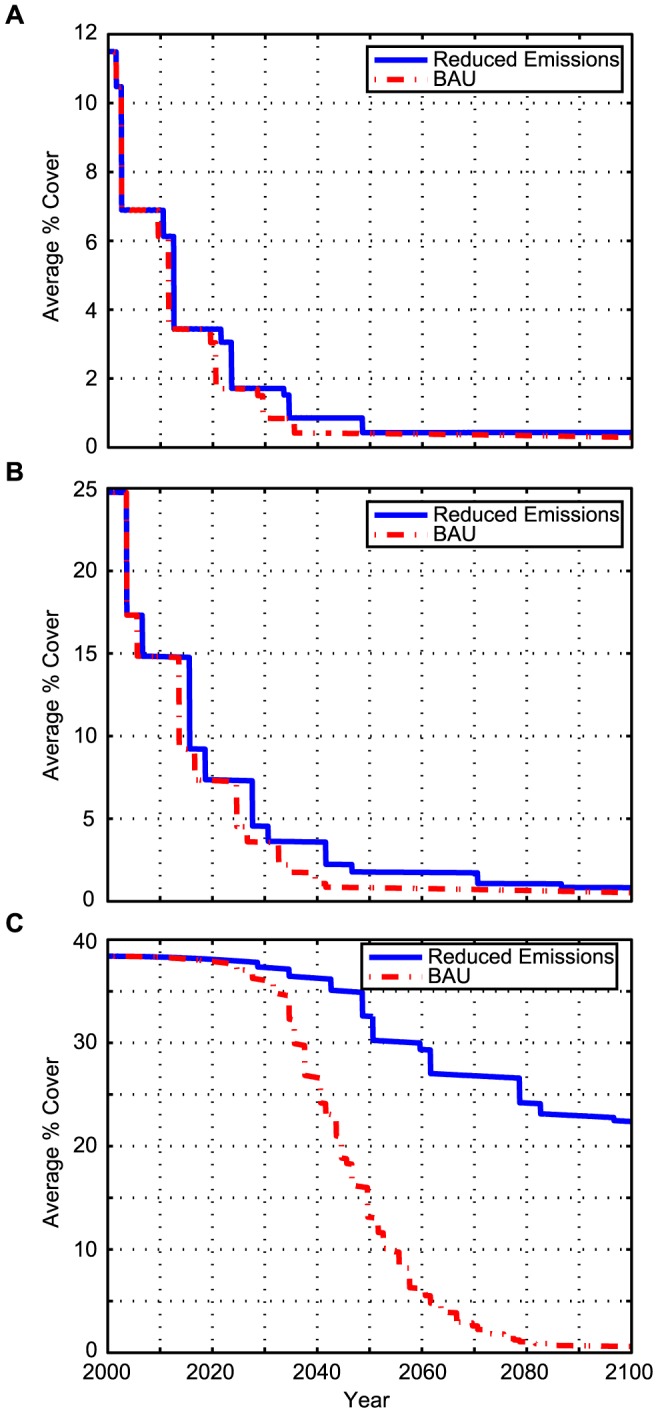
a–c. Coral cover for two climate scenarios in a) Florida, b) Puerto Rico, and c) Hawaii.

In COMBO, changes in coral cover are affected by both the sharp declines in cover that occur after episodic bleaching events and by long-term changes in coral cover associated with elevated temperatures and increasing CO_2_ concentrations. Figure 1 depicts changes in coral cover (averaged across grid cells) for the policy and BAU scenarios for each of the three locations.

In South Florida, initial average coral cover is 11.5%, but is projected to drop to below 1% cover under both the reduced emissions and BAU scenarios by 2040 (Figure 1a). In Puerto Rico, initial average coral cover is 24.8%, but is projected to drop to 1.1% under the BAU scenario and 3.6% under the reduced emissions scenario by 2040 (Figure 1b). In Hawaii, initial average coral cover is 38.4%, but is projected to drop to 27% under the BAU scenario and 36% under the reduced emissions scenario by 2040 (Figure 1c). In Hawaii, projected differences in average coral cover between the scenarios begin in approximately 2025 and increase steadily, with coral cover dropping more rapidly for the BAU scenario (Figure 1c).

In Hawaii, temperature-induced bleaching events are the dominant factor leading to the loss of coral cover, but ocean acidification also contributes to coral cover loss. Under the reduced emissions scenario, coral cover is projected to be 22.4% in 2100 when acidification is included in the model compared to 24.5% when the impacts of acidification are excluded (not shown in Figure 1c). In South Florida and Puerto Rico, initial coral cover is low and declines quickly enough that the separate effect of acidification on coral cover is negligible.

### 3.3 Economic Valuation Results

As coral cover declines, so will the values generated by the coral resource. The magnitude of the decline depends on both the trajectory of coral cover loss and initial starting values in each location. Under both the reduced emissions scenario and the BAU scenario, coral values are projected to decline over the 21st century, compared to current values. Here, we present the loss of value that is projected to be avoided if policies leading to reduced emissions were implemented instead of the world continuing on a BAU trajectory.

**Figure 2:**
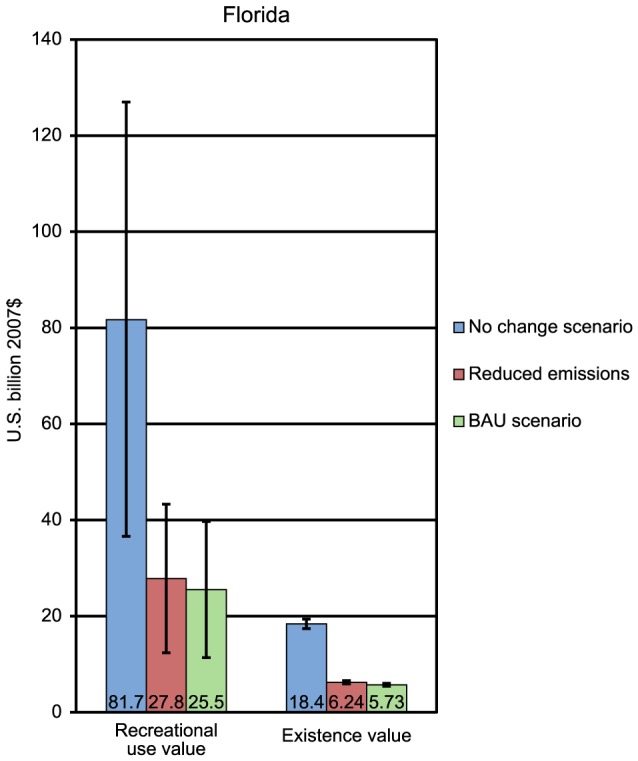
Economic values (with 95% confidence interval) for BAU and reduced emission scenarios for Florida.

**Figure 3:**
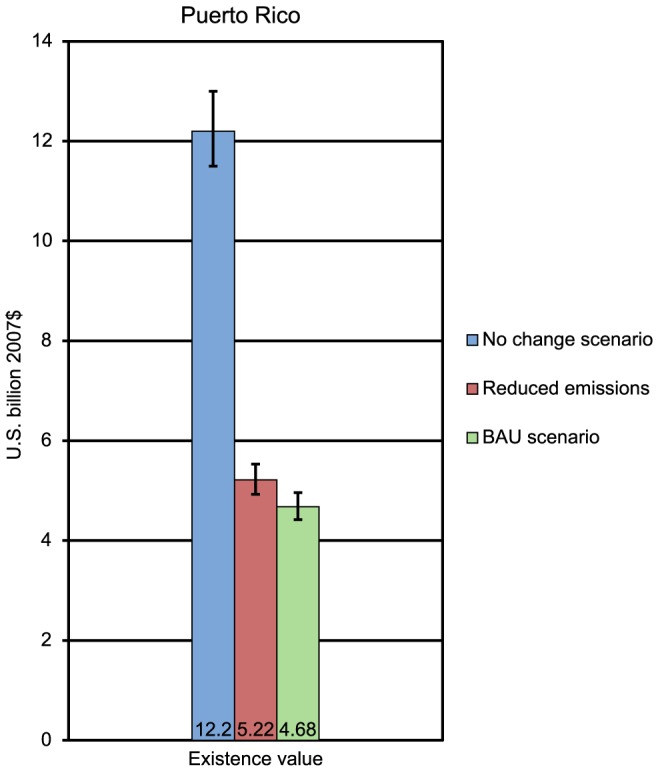
Economic values (with 95% confidence interval) for BAU and reduced emission scenarios for Puerto Rico.

**Figure 4:**
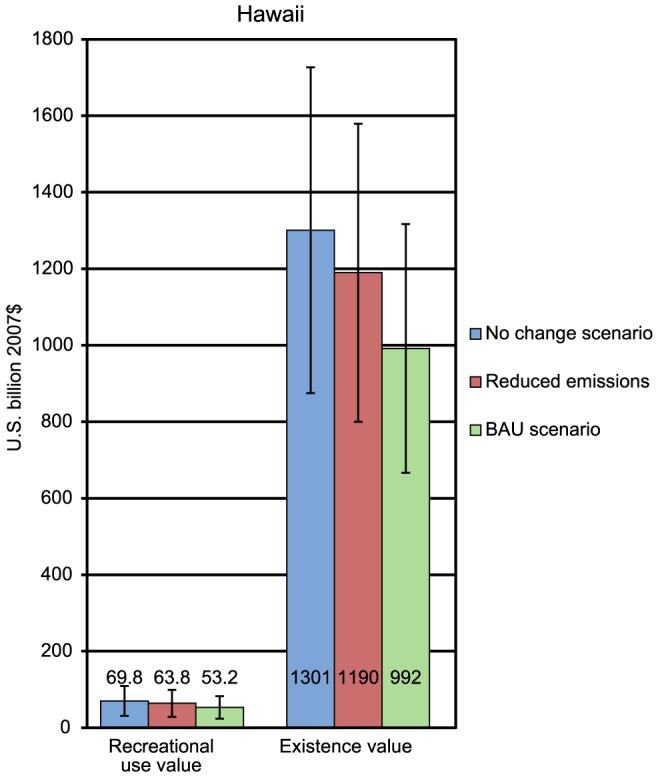
Economic values (with 95% confidence interval) for BAU and reduced emission scenarios for Hawaii.

For Florida, compared to the BAU scenario, the reduced emissions scenario results in an estimated avoided loss with a discounted present value of approximately $2.29 billion in recreational use values and $0.46 billion in existence values (Figure 2). In Puerto Rico, the reduced emissions scenario results in an estimated avoided loss of $546 million in existence values compared to the BAU scenario (Figure 3). In Hawaii, the reduced emissions scenario results in an estimated avoided loss with a present value of approximately $10.6 billion in recreational use values and $0.59 billion in existence values compared to the BAU scenario (Figure 4). It is worth remembering that existence values in all three locations exclude nonresidents.

The preceding paragraph contrasts the benefits generated by reefs under the BAU scenario to the benefits generated under the policy scenario. It is also instructive to compare the benefits generated by reefs in the future to the benefits currently being enjoyed. For each region, the present value of future reef benefits under each scenario was compared to what those future reef benefits would have been without any decline in reef cover. For both Florida and Puerto Rico, the percent decline in reef values is similar under the BAU and the policy scenarios. For Florida, the BAU scenario is associated with a 69% decrease in present value, while the policy scenario is associated with a 66% decrease in present value. For Puerto Rico, the BAU scenario is associated with a 62% decrease in present value while the policy scenario is associated with a 57% decrease. In contrast, in Hawaii, the BAU scenario is associated with a 24% decrease in present value, while the policy scenario is associated with a 9% decrease in present value.

## Discussion

### 4.1 Key Uncertainties and Factors Not Included in the Model

There are important uncertainties in the ecological and economic assumptions and data used to develop the model, in part deriving from factors that are not included in the model. These could cause the model to produce either high or low estimates of changes in coral cover and the associated declines in economic value. Additional uncertainties regarding the COMBO model were discussed in a previous COMBO study [20].

Ecological conditions and biological mechanisms could lead to higher or lower levels of coral cover compared to the projections provided here. In addition to the factors affecting coral cover, other factors can directly affect the magnitude and time course of the related economic losses estimated here. Because there is little information available to quantify most of the sources of uncertainties, we present a list of factors, the probable direction of their effect (i.e., increase or decrease of loss compared to the results of this analysis), and a very approximate estimate of relative magnitude in Table 6.

**Table 6:** Likely effects of uncertainty factors on ecological and economic values of coral reefs, compared to model projections for BAU and policy scenarios^a^.

Factors not currently included in the COMBO model	Likely effect on coral cover	Likely effect on economic values provided by coral reefs	Notes
1. Long-term adaptive capacity of corals	Increase	Increase	Adaptation will occur, but too slowly for major beneficial effect for coral reefs compared to timing of bleaching events
2. Inclusion of deep reefs that are less susceptible to bleaching	Increase	Increase	Contribution to sustainability and to coral reef values not included in model; recreational values of deep reefs are likely to be lower than recreational values of more accessible, shallow reefs
3. Variable coral response to stressors	Increase	Increase	Selective survival of different coral types may slow loss of cover but would not affect loss of diversity
4. Additional sources of mortality	**Decrease**	**Decrease**	Intensification of existing non-climate factors, addition of factors not currently present in an area (e.g., increased runoff), and/or synergism of existing factors would decrease coral cover
5. Additional impacts of ocean acidification to the ocean ecosystem	Decrease	Decrease	Potentially important for decreasing cover, especially in Hawaii, but probably a longer-term impact than temperature effects
6. Additional declines in baseline growth rate and/or increases in baseline mortality rate	**Decrease**	**Decrease**	Certain to occur and almost certainly detrimental for coral cover
7. Impacts of climate change and acidification on reproduction/recruitment	Decrease	Decrease	Likely to occur, but impacts of coral mortality and morbidity will dominate over reproductive effects
8. Improved coastal management, conservation	Increase	Increase	May occur in some locations, but general trend has been greater impacts from coastal management, rather than improvement
9. Economic reef values different from values used		**Increase** or decrease	Value estimates used in the modeling are likely skewed to the low side (not all affected population)
10. Value is a nonlinear function of cover		Increase or decrease	Depends on the cover value of the tipping point
11. Inclusion of other ecosystem services not analyzed in our model		**Increase**	Coastal protection, fisheries, and larger ecosystem interactions were neglected
12. Variations of modeled climate sensitivity other than 3°C	Increase under lower values and **decrease** under higher values	Increase under lower values and **decrease** under higher values	Caribbean results are unlikely to change, however, higher sensitivity values could result in greater Hawaiian damages.

^a^ Factors judged likely to have a substantially greater impact than others are indicated by bold type.

Table 6 indicates that the preponderance of the uncertainty considerations suggest that this study is likely to underestimate future coral reef cover loss and economic damages. For example, the COMBO model does not incorporate any density-dependent effects of reduced coral cover on coral survivorship or growth rates. In contrast, a recent study in Puerto Rico [50] found that the 2005 mass-bleaching event led to increased fragmentation of coral populations, with reduced survivorship and recruitment for small populations after bleaching.

One of the higher importance factors noted in Table 6 is the additional sources of coral mortality. A previous study of global coral reef health [3] found that more than 75% of reefs in the Atlantic/Caribbean region were affected by local threats (e.g., coastal development, marine-based pollution and damage, overfishing, or watershed-based pollution), all of which can affect coral health and coral cover. Thus, coral cover losses, under both the BAU and policy scenarios, are likely to be even greater than the levels predicted here based on climate change impacts only. In contrast, in the Pacific Region (including Hawaii), fewer than 50% of reefs were classified as affected by local threats, suggesting a greater importance of climate change impacts on coral reef cover and value.

Because Hawaii has considerably longer persistence of reefs in the COMBO projections, especially under the reduced emissions scenario, factors that act over the longer-term may have greater importance there than in South Florida and Puerto Rico. In particular, factors 1 (adaptive capacity), 5 (broader acidification impacts), and 7 (impacts on reproduction/recruitment) from Table 6 might have greater impacts on the Hawaiian reefs later in this century These factors would not, however, change the combined uncertainties enough to alter the general conclusions. Factor 12 in Table 6 (variations in climate sensitivity) would potentially reduce the benefit of the reduced emissions scenario compared to the BAU scenario if the climate sensitivity was much higher than the 3°C assumed here (e.g., 6°C); under a high climate sensitivity, the reduced emissions scenario may not sufficiently mitigate increased temperatures to allow Hawaiian reefs to persist to the end of the 21st century. In addition, we assume that coral reef values on a per-unit basis would stay constant over the next century. As coral reefs become scarcer and incomes rise in the developing world, it is likely that coral reef values may increase on a per-unit basis [51], even as total coral reef values may decline because of degradation and loss of coral cover. Thus, our analysis of economic benefits may understate the relative benefits of scenarios that preserve coral reef cover, as coral reefs become scarcer.

### 4.2 Ecological and Economic Outcomes under the Reduced Emissions and BAU Scenarios

The modeling results presented here suggest that future prospects for coral reef communities and the services and economic values they provide are bleak for South Florida and Puerto Rico, regardless of whether global action is taken to mitigate climate change. The projected GHG mitigation actions associated with the reduced emissions scenario appear insufficient to avoid multiple bleaching and mortality events in the next 30 years in South Florida and the Caribbean (as represented by Puerto Rico). For these areas, the COMBO model projects three significant bleaching events by 2020 under both the reduced emissions and BAU scenarios. At the percent cover projected for 2020, we suggest that shallow reefs in these areas would be better viewed as shallow-water ecosystems that contain individual coral organisms, rather than sustainable structural ‘coral reef’ communities. It will be very difficult for reefs with coral cover <5–10% to have a net positive reef development associated with local recruitment and the accumulation of endogenous carbonate (e.g., [52]). Once established, the downward trajectory of coral cover is likely to continue. However, the reduced emissions scenario (and its associated lower SSTs and reduced ocean acidification) may provide benefits to localized areas of more resilient corals that can resist bleaching events to a greater extent.

Our valuation estimates for South Florida are comparable to another study that projected declines in coral cover and the associated economic value if no actions are taken to mitigate the environmental risks to coral [2]. For South Florida, if the discounted loss in recreational use value under the BAU scenario were spread out equally among years, it would amount to roughly $560 million of lost economic value per year compared to the no-change scenario. As a comparison, a different study conducted prior to the major 2005 bleaching event in the Caribbean suggested that continued coral reef degradation in the Caribbean could reduce the recreational use value benefits derived from these ecosystems by a comparable $350–850 million per year [2].

In Hawaii, however, where temperatures are cooler, current levels of coral cover are higher, and more warming is required to initiate bleaching, the reduced emissions scenario results in a projection of significant ecological benefits for coral reefs. The reduced emissions scenario leads to avoided bleaching in some areas, delayed bleaching in other areas, and higher and more persistent levels of coral cover. As discussed previously, the reduced emissions scenario is associated with an economic ‘savings’ (compared to a BAU scenario) of $10.6 billion in recreational use values and $0.59 billion in existence values over 100 years.

There is considerable uncertainty surrounding these projected values. Indeed, the National Research Council noted in a 2009 report on the costs of climate change that uncertainty and information limitations pose difficulties to any analysis of such complex and long-term impacts [53]. We acknowledge these uncertainties and limitations but view the economic value estimates presented here as more informative than the default assumption that coral reefs have zero value. Accordingly, the results presented in this paper support ongoing efforts to develop best possible estimates of these ecosystem services. In particular, the development of benefit metrics helps agencies analyze the anticipated economic effects of proposed standards and policies and comply with rulemaking guidelines for such assessments. Consideration of the limitations and uncertainties suggests several reasons that the value is likely to be an understatement of the potential benefit associated with implementing the reduced GHG emissions scenario compared to the BAU scenario for Hawaiian reefs. Specifically, a full accounting of the existence values associated with saving coral reefs in Hawaii is likely to be higher than the existence estimate calculated here. For our analysis, we took a benefit transfer approach and applied the per-capita existence value for avoiding coral reef loss from Puerto Rico to the population in Hawaii. However, if this value was applied to the whole U.S. population (as was done for a separate study of existence values associated with improving reef quality in Hawaii [33]), our estimate of the increase in existence values associated with the reduced emissions scenario would increase from $0.59 billion to more than $140 billion.

### 4.3 Management Implications of Findings

Our study shows a contrast in modeling outcomes between those for South Florida and Puerto Rico and those for Hawaii. In South Florida and Puerto Rico, the model projects modest benefits associated with the reduced emissions scenario relative to the BAU scenario, but these benefits are projected to occur after coral reefs are likely to have been decimated already by multiple, severe bleaching events in either scenario. The threats posed by climate change suggest that local management actions to increase reef resiliency to bleaching are critical for reducing coral loss. In the 2004 *Reefs at Risk in the Caribbean* report, over 80% of Puerto Rico's reefs were rated as high risk, while in Florida, over 60% were rated as threatened [2]. Local management recommendations that would benefit reefs include reducing unsustainable fishing that leads to the loss of grazers that control algal growth on reefs; managing coastal development and reducing watershed-based pollution to reduce the impacts of erosion, nutrient runoff, and sewage; reducing marine-based pollution and damage; and reducing the impact of tourism that can physically damage corals [3]. In Puerto Rico, the lack of laws regulating fishing and recreation limits effective management, while in Florida, heavy recreational use and high levels of tourism contribute to overfishing, physical damage, and sewage impacts [2].

In Hawaii, there is still substantial opportunity for emissions reductions to avoid or delay bleaching and maintain higher levels of coral cover. Only 17% of Hawaiian reefs were rated as threatened in the most recent *Reefs at Risk: Revisited* analysis [3]. Local management actions, including managing coastal development and targeting invasive species which can harm reefs, such as several species of invasive algae, can benefit corals [3]. A combination of local management actions and emissions reductions will help maintain the economic values of coral reefs that provide significant benefits to the island population whose livelihoods depend upon coral reefs, the tourists who value the recreational experiences available on coral reefs, and the general population that values coral reefs for their beauty, diversity, or the critical role that reefs play in sustaining healthy ocean and coastal ecosystems.
